# Spontaneous Rupture of a Right Gonadal Artery Aneurysm: A Case Report

**DOI:** 10.7759/cureus.57352

**Published:** 2024-03-31

**Authors:** Krystal T Nguyen, Austin Henken-Siefken, Robert Fincher, Andrew McCague

**Affiliations:** 1 Clinical Sciences, Western University of Health Sciences, Pomona, USA; 2 Surgery, Desert Regional Medical Center, Palm Springs, USA; 3 Trauma, Desert Regional Medical Center, Palm Springs, USA; 4 Trauma and Acute Care Surgery, Desert Regional Medical Center, Palm Springs, USA

**Keywords:** rupture, embolization, pseudoaneurysm, visceral artery aneurysm, ovarian artery aneurysm, gonadal artery aneurysm

## Abstract

Gonadal artery aneurysm is a rare condition characterized by nonspecific presentation, typically manifesting as flank pain and formation of a retroperitoneal hematoma on imaging studies. Failure to recognize and treat this condition promptly can have serious consequences, as the presence of an aneurysm may lead to severe bleeding. Notably, most reported cases of gonadal artery aneurysms are not trauma-induced but rather spontaneous. In this case report, we describe the case of a previously healthy woman in her late 30s who presented to the emergency department with initial symptoms of flank pain and elevated white blood cell count. Subsequent imaging via computed tomography of the abdomen and pelvis revealed a significant hematoma surrounding the right kidney, indicative of a substantial hemorrhagic event. Angioembolization followed by endograft placement was performed on the patient, and she was expected to make a full recovery.

## Introduction

Gonadal artery aneurysm is a rare condition that occurs due to a tear in the vessel, resulting in the formation of a retroperitoneal hematoma [1]. The gonadal artery originates from the anterior aspect of the aorta and runs alongside the psoas major muscle, in front of the ureters and external iliac arteries. In females, it supplies the ovaries and the distal third of the fallopian tube. In males, it supplies the testes and epididymis as it becomes the testicular artery. The clinical presentations of aneurysms are often nonspecific, characterized by flank pain and retroperitoneal hematoma, thus requiring clinicians to recognize these symptoms and provide timely intervention to avoid severe complications. In this clinical case, our patient had a gonadal artery aneurysm that was treated with angioembolization, followed by stent graft placement. By sharing this case, we hope to contribute to the understanding and management of gonadal artery aneurysms, highlighting the importance of prompt diagnosis and appropriate treatment.

## Case presentation

A 39-year-old female, G0P0, presented to the emergency department with a one-day onset of right flank pain. The initial evaluation showed she was hypotensive at 75/56 mmHg and tachycardic with a heart rate of 103 beats/minute and a respiratory rate of 18 breaths/minute. She was non-febrile at 36.7°C. Workup pointed towards septic shock based on her initial labs showing a white blood cell count of 24,000 cells/μL, hemoglobin level of 11.9 g/dL, hematocrit of 35.9%, platelet count of 414,000/mm^3^, sodium of 137 mEq/L, potassium of 4.5 mEq/L, chloride of 103 mEq/L, carbon dioxide level of 21 mmHg, glucose of 215 mg/dL, blood urea nitrogen of 13 mg/dL, creatinine of 1.1 mg/dL, lactic acid of 1.96 mg/dL, alkaline phosphatase of 63 U/L, alanine aminotransferase (ALT) of 18 U/L, aspartate aminotransferase (AST) of 29 U/L, lipase of 99 U/L, and total bilirubin of 1.7 mg/dL.

**Figure 1:**
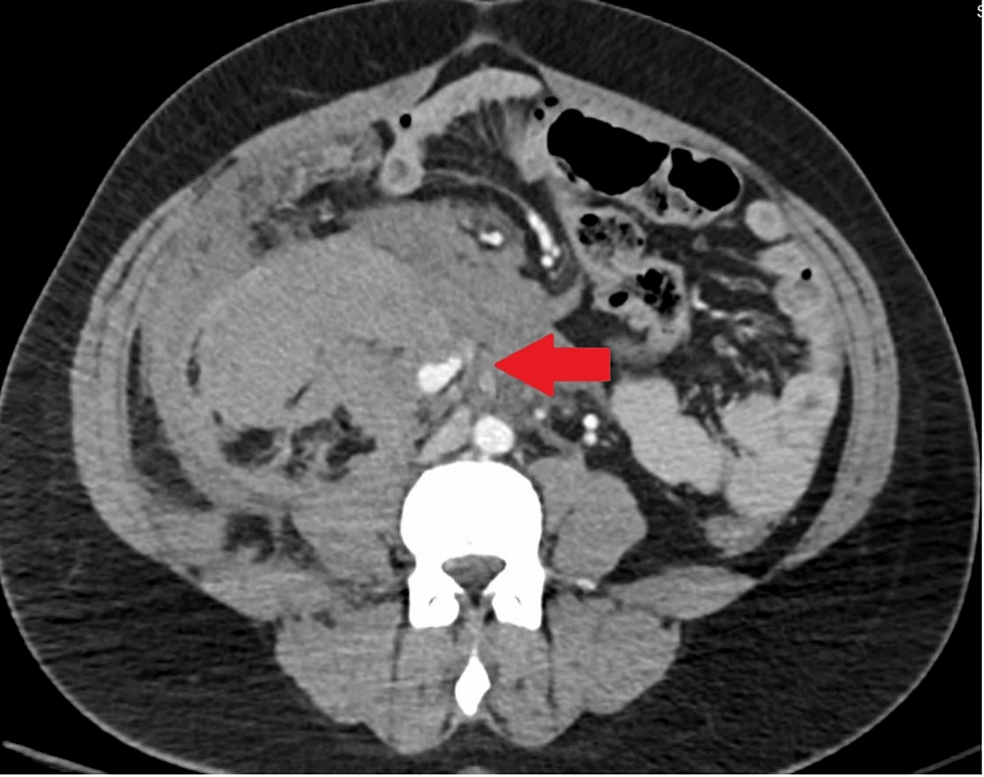
Right gonadal artery aneurysm with surrounding hematoma suggesting rupture. The arrow points to the aneurysm.

**Figure 2:**
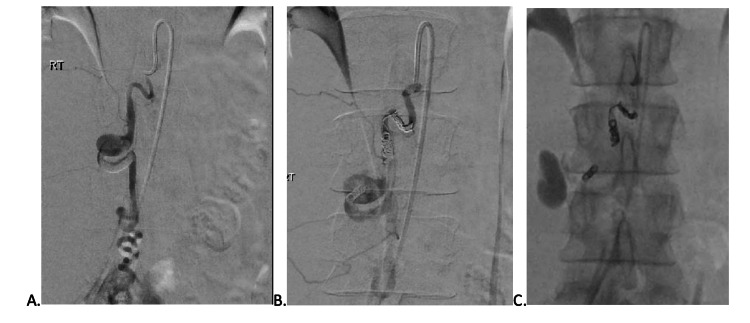
Spontaneous right gonadal artery aneurysm before and after treatment. (A) Right gonadal artery aneurysm before angiogram. (B) After coiling. (C) Delayed image showing extravascular at the level of the aorta.

A computed tomography (CT) of the abdomen and pelvis was performed for additional workup, which showed a large amount of hemorrhage within the right retroperitoneum encasing the right kidney. The hemorrhage occurred along the right gonadal artery which had an area of focal dilated tubular-shaped structure concerning for congenital artery aneurysm with subsequent rupture (Figure 1). The aneurysm is likely congenital or related to a connective tissue disorder, given the absence of trauma or infection and the patient has no history of being pregnant. Interventional radiology was later consulted for angioembolization, where a partially thrombosed right gonadal artery aneurysm measuring 9.4 mm in diameter was revealed. After angioembolization, a CT angiography (CTA) was done showing occlusion at the right gonadal artery, with concerns for occlusion or possible avulsion. Vascular was consulted, and this patient underwent emergent endograft placement over the area of the right gonadal artery takeoff. After endograft placement, no additional extravasation was seen (Figure 2). The patient will be worked up outpatient for connective tissue disorders which may be a cause of her aneurysms and bleeding.

## Discussion

Gonadal artery aneurysm is a relatively rare condition that can be life-threatening if left untreated. In the last 20 years, less than 30 cases of this condition have been reported [1-6]. Most cases are related to pregnancy or premenopausal peripartum period, although cases occurring during postmenopausal have also been documented [7]. Bilateral gonadal artery aneurysms are even more rare, with only one reported case [6]. It is important to note that most of the reported cases have been spontaneous.

Gonadal artery aneurysms are uncommon, but they can and often occur during pregnancy or during the peripartum period [8,9]. These aneurysms may develop as a result of anatomical and physiological changes associated with pregnancy as the artery often gets dilated to accommodate for increased blood flow [1,3,8,9]. Repetitive pregnancies have also been suggested to cause a loss in arterial wall integrity, increasing susceptibility to aneurysms or ruptures [6]. When evaluating patients presenting with flank pain or abdominal pain resulting in periarterial hematoma formation, the goal is to identify the source of bleeding and perform necessary interventions. In cases of trauma, a trauma workup is essential. However, most cases of gonadal artery aneurysm occur without preceding trauma. In the absence of trauma, pregnancy-related causes should be considered, especially in reproductive-age females.

The initial workup should include a thorough history and physical examination, including past and current obstetric history, vital signs, vaginal bleeding, fluid leakage, and abdominal examination. Gastrointestinal disorders and ovarian torsion should be ruled out. Patients with vomiting or epigastric pain should be evaluated for pregnancy-related disorders. Delays in the diagnosis and treatment of pregnant women can increase the risk of adverse maternal and fetal outcomes. Hypotension, as seen in this case and most reported cases, can be a sign of sepsis or hemorrhage, and intraabdominal infection can certainly lead to sepsis. In the absence of trauma, rupture of a blood vessel should be considered in the differential.

Ultrasonography is commonly used as the first-line diagnostic tool in the emergency department, especially for pregnant women. Other diagnostic modalities such as CT and magnetic resonance imaging (MRI) depend on the hemodynamic stability of the patient, availability, and risk of radiation exposure. While MRI provides better imaging without ionizing radiation exposure, its limited availability and high cost make it less accessible.

Although surgical intervention is an option, most reported cases have been successfully treated with angioembolization. In this particular clinical case, the patient was treated with coil and Gelfoam embolization, followed by intermittent extravasation during the procedure. Due to the need for extensive exposure to locate the bleeding source, an endovascular technique was chosen instead of an open procedure. When a post-embolization CTA revealed occlusion at the right gonadal artery, an endograft was placed over the area where the right gonadal artery originates to address concerns of occlusion or possible avulsion. The exact cause of the additional bleeding occurring post-angioembolization is unclear; however, it is suspected that it may be a result of injury to the vessel when removing the wires after coiling, a common complication associated with interventional radiology procedures.

Angiography with embolization has replaced open surgery as the treatment of choice for these lesions by most surgeons due to its minimally invasive nature. Both approaches show no statistically significant difference in mortalities; however, the endovascular approach with refined catheter access, embolization technique, and stent graft technique offers advantages such as less postoperative pain, fewer complications, decreased length of hospital stay, and shorter recovery time [10]. Therefore, it is reasonable to consider endovascular treatment as the preferred first line for management. However, in cases where endovascular options are not reasonable, open surgery remains a viable alternative to treat these aneurysms. Furthermore, an additional consideration regarding embolization is the potential for re-extravasation when the substances used are absorbed, thus raising concerns about the material utilized during the embolization procedure [2].

## Conclusions

Gonadal artery aneurysm is a relatively rare condition. The initial presentation of this condition can be mistaken for other conditions, such as sepsis or kidney-related problems, if based solely on lab workup or clinical assessment. Women of reproductive age and those in the postpartum period who present with abdominal or flank pain, leukocytosis, and hemodynamic instability should have gonadal artery rupture or aneurysm on the differential.
